# A genetic screen to uncover mechanisms underlying lipid transfer protein function at membrane contact sites

**DOI:** 10.26508/lsa.202302525

**Published:** 2024-03-18

**Authors:** Shirish Mishra, Vaishnavi Manohar, Shabnam Chandel, Tejaswini Manoj, Subhodeep Bhattacharya, Nidhi Hegde, Vaisaly R Nath, Harini Krishnan, Corinne Wendling, Thomas Di Mattia, Arthur Martinet, Prasanth Chimata, Fabien Alpy, Padinjat Raghu

**Affiliations:** 1 National Centre for Biological Sciences -TIFR, GKVK Campus, Bangalore, India; 2 School of Biotechnology, Amrita Vishwa Vidyapeetham, Kollam, India; 3 Université de Strasbourg, CNRS, Inserm, IGBMC UMR 7104- UMR-S 1258, Illkirch, France

## Abstract

Lipid transfer proteins mediate the transfer of lipids between organelle membranes, and the loss of function of these proteins has been linked to neurodegeneration. However, the mechanism by which loss of lipid transfer activity leads to neurodegeneration is not understood. In *Drosophila* photoreceptors, depletion of retinal degeneration B (RDGB), a phosphatidylinositol transfer protein, leads to defective phototransduction and retinal degeneration, but the mechanism by which loss of this activity leads to retinal degeneration is not understood. RDGB is localized to membrane contact sites through the interaction of its FFAT motif with the ER integral protein VAP. To identify regulators of RDGB function in vivo, we depleted more than 300 VAP-interacting proteins and identified a set of 52 suppressors of *rdgB*. The molecular identity of these suppressors indicates a role of novel lipids in regulating RDGB function and of transcriptional and ubiquitination processes in mediating retinal degeneration in *rdgB^9^*. The human homologs of several of these molecules have been implicated in neurodevelopmental diseases underscoring the importance of VAP-mediated processes in these disorders.

## Abstract (precis)

A genetic screen in *Drosophila* photoreceptors uncovers multiple regulators of lipid transfer function and endoplasmic reticulum–plasma membrane contact sites.

## Introduction

The maintenance of exact membrane lipid composition is important for providing a distinct identity to cellular organelles and thus for supporting normal cellular physiology (Harayama & Riezman, 2018). Various lipid species reach their specific organelle membrane via either vesicular or non-vesicular transport. Proteins that shuttle lipids in a non-vesicular manner across various compartments are known as lipid transfer proteins (LTPs). Each of these LTPs transfer specific lipid species such as sterols, ceramides, or phospholipids, and in many cases, the LTPs are localized at very specific locations known as membrane contact sites (MCS). In a eukaryotic cell, MCS are regions where two organelle membranes come very close at the range of 10–30 nm but do not fuse (Prinz et al, 2020). Being the largest cellular organelle, the ER forms MCS with the mitochondria, lysosomes, the Golgi network, lipid droplets, and the plasma membrane (PM). MCS provide the fast and efficient delivery of metabolites between two membranes and could be permanent or induced (Wu et al, 2018); this includes the exchange of lipids between organelle membranes to support ongoing cell physiology (Cockcroft & Raghu, 2018). Growing evidence suggests an important role of LTP function at MCS and LTPs in human neurological disorders (Fowler et al, 2019; Peretti et al, 2020; Guillén-Samander & De Camilli, 2022). However, much remains to be discovered on the regulation of LTP function at MCS.

MCS between the ER and the PM are important for regulating both plasma membrane lipid composition and signalling functions. One of the best examples of the requirement of an LTP at the ER-PM MCS is sensory transduction in *Drosophila* photoreceptors (Yadav et al, 2016). Photoreceptors detect light through the G protein–coupled receptor rhodopsin (Rh), leading to the hydrolysis of phosphatidylinositol 4,5-bisphosphate [PI(4,5)P_2_] by G protein–coupled PLC activity (Hardie & Raghu, 2001). As part of their ecology, fly photoreceptors are exposed to light; in bright daylight, they typically absorb ca. 10^6^ effective photons/second resulting in extremely high PLC activity. Hence, fly photoreceptors provide an excellent model system to study the turnover of PI(4,5)P_2_ during PLC-mediated cell signalling (Raghu et al, 2012).

Given the low abundance of PI(4,5)P_2_, replenishment of this lipid at the PM is necessary for uninterrupted PLC signalling. Many enzymes and proteins participate in this process, but a key step is the transfer of lipids that are intermediates of the PI(4,5)P_2_ cycle. One of the proteins at this site is retinal degeneration B (RDGB), a large multi-domain protein with an N-terminal phosphatidylinositol transfer protein (PITP) domain (Raghu et al, 2021). The PITP domain belongs to the superfamily of LTPs. In the case of RDGB, its PITP domain can transfer phosphatidylinositol (PI) and phosphatidic acid (PA) in vitro (Yadav et al, 2015); a property that is conserved in its mammalian ortholog, Nir2 (Kim et al, 2015). *rdgB* mutant flies undergo light-dependent retinal degeneration, a reduced electroretinogram (ERG) response, and a reduced rate of PI(4,5)P_2_ resynthesis at the PM after PLC activation (Hotta & Benzer, 1970; Harris & Stark, 1977; Yadav et al, 2015). In photoreceptors, RDGB is localized at the ER-PM MCS formed between the microvillar plasma membrane and the SMC, a specialization of the smooth ER (Yadav et al, 2016). The localization of RDGB at this MCS is critically dependent on its interaction with the ER integral membrane protein, VAP (vesicle-associated membrane protein–associated protein). This interaction is physiologically relevant as disruption of the protein–protein interaction between RDGB and VAP in *Drosophila* photoreceptors results in mislocalization of RDGB from this MCS, reduces the efficiency of PI(4,5)P_2_ turnover, and impacts the response to light (Yadav et al, 2018). However, the mechanisms by which the activity of RDGB is regulated by other proteins at the MCS in this in vivo model system remain to be discovered. VAPs are involved in a range of interactions with proteins containing FFAT/FFNT/Phospho-FFAT/non-FFAT motifs (Slee & Levine, 2019; Cabukusta et al, 2020; Di Mattia et al, 2020). Thus, it seems possible that other proteins involved in regulating biochemical activity at this MCS might also be localized to the sub-microvillar cisternae via VAP interactions. The identification and analysis of proteins engaged in VAP-dependent interactions might help in understanding the regulation of RDGB function. Importantly, VAPs have been implicated in neurodegenerative disorders such as amyotrophic lateral sclerosis, frontotemporal dementia, Alzheimer’s disease (AD), and Parkinson’s disease (reviewed in Dudás et al [2021]).

In this study, we have carried out a proteomics screen to identify protein interactors of VAP-A and VAP-B in mammalian cells and tested their functional significance in the context of neurodegeneration using the experimental paradigm of RDGB function in *Drosophila* photoreceptors in vivo. The candidates so identified perform a wide range of sub-cellular functions indicating an extensive network of biochemical processes that control the function of RDGB in regulating lipid transfer during PLC signalling, thus maintaining the structural and functional integrity of neurons.

## Results

### Strategy of proteomics screen

**Figure 1.**
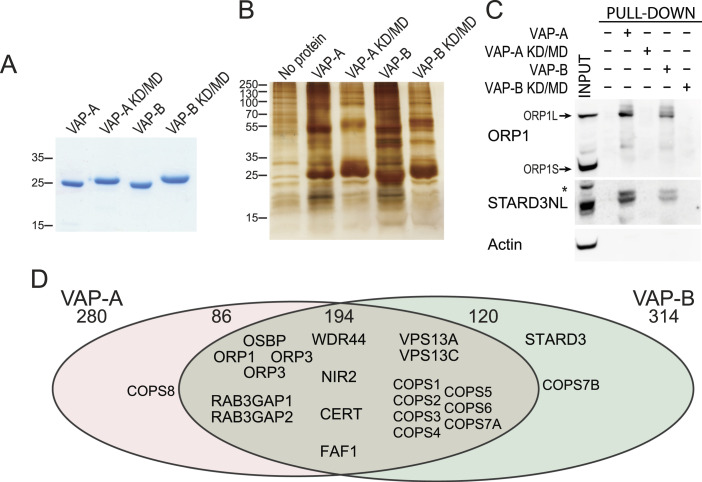
Identification of VAP-A and VAP-B binding partners. **(A)** Coomassie Blue staining of the recombinant WT and KD/MD mutant MSP domains of VAP-A and VAP-B after SDS–PAGE. **(B)** Silver nitrate staining of proteins pulled down using WT MSP domains of VAP-A and VAP-B, and the KD/MD mutant MSP domains, after SDS–PAGE. **(C)** Western blot analysis of proteins pulled down using the WT and mutant MSP domain of VAP-A and VAP-B. The input and pull-down fractions correspond to HeLa cell total protein extract and bound proteins, respectively. *: non-specific band. **(D)** Venn diagram of proteins pulled down by VAP-A and VAP-B (and not by mutant VAP-A and VAP-B). A total of 401 proteins were pulled down with either VAP-A or VAP-B. 194 proteins were pulled down with both VAP-A and VAP-B.

To obtain a list of proteins interacting with VAPs, we performed pull-down experiments in human cells. We produced, in *Escherichia coli*, and purified the MSP domain of human VAP-A and VAP-B fused to a C-terminal 6His-tag (Fig 1A). As a negative control, we used the K94D/M96D and K87D/M89D mutants (herein named KD/MD mutants) of VAP-A and VAP-B, respectively, that are unable to bind FFAT (two phenylalanines in an acidic tract) motifs (Kaiser et al, 2005; Wilhelm et al, 2017). Recombinant proteins were attached to a Ni^2+^-NTA resin and then incubated with protein extracts from HeLa cells. Bound proteins were eluted and analysed by SDS–PAGE followed by silver nitrate staining (Fig 1B) that showed numerous differential bands between WT and mutant VAP samples, suggesting that many proteins are pulled down owing to VAP’s ability to bind FFAT motifs. To verify the pull-down efficiency, we performed Western blot using antibodies against two known VAP partners, ORP1 and STARD3NL (Fig 1C) (Rocha et al, 2009; Alpy et al, 2013). ORP1 exists as a long and a short isoform called ORP1L and ORP1S, respectively, ORP1L being the only one of the two to possess an FFAT motif. As expected, the ORP1L isoform was pulled down by WT VAPs but not by mutant VAPs, and the ORP1S isoform was not precipitated (Fig 1C). Besides, STARD3NL co-precipitated with WT VAP-A and VAP-B and not with mutant VAPs, whereas actin, used as a loading control, was not found in the eluted fractions (Fig 1C and Table S1). To identify the proteins pulled down by VAPs, eluates were analysed by tandem mass spectrometry (MS/MS) (referred to below as IP-MS). The full list of interactions identified by MS/MS in this experiment is shown in Table S2. To identify proteins pulled down according to their ability to interact with VAPs in an FFAT-dependent manner, proteins were ranked based on their enrichment in the WT over the KD/MD mutant VAP sample, and on their MS/MS score (Fig 1D). This strategy led to the identification of 401 proteins, 194 of which were pulled down by both VAP-A and VAP-B. Interestingly, many known partners of VAP-A and VAP-B, such as OSBP, ORP1, ORP2, WDR44, VPS13A, and VPS13C, were identified (Fig 1D). Using a position weight matrix strategy, we looked for potential FFAT and Phospho-FFAT in the protein sequences; sequences were attributed a score, with 0 corresponding to an ideal FFAT/Phospho-FFAT sequence. Among the 401 proteins identified, 136 had an FFAT or Phospho-FFAT with a significant score (between 0 and 2.5) (Table S1). We used this list of 401 mammalian proteins and identified their *Drosophila* orthologs using DRSC Integrative Ortholog Prediction Tool (Hu et al, 2011), and the fly orthologs with the best score were identified. Using this approach, we were able to identify fly orthologs with more than 90% coverage for 393 of 401 mammalian proteins in the VAP interaction list (Table S3).

Table S1. Proteins identified by MS/MS after VAP-A and VAP-B pull-down and interacting in an FFAT-dependent manner. For each protein, the UniProt ID, description, and Ratio Score corresponding to the ratio of PSMs (peptide–spectrum matches) obtained with the WT and KD/MD mutant VAP are indicated (see Table S2). Moreover, the score, position, and sequence of the two best conventional FFAT and Phospho-FFAT sequences are shown. Proteins identified in both VAP-A and VAP-B pull-down are labelled with a green background (columns A-B), and proteins identified in BioGRID 4.4.223 (Oughtred et al, 2019) as VAP partners are labelled in cyan (column AC). FFAT scores are colour-coded with a scale from orange to blue (dark to light orange: 0–3; light to dark blue: 3.5->5). Acidic, phosphorylatable (S, T only), and aromatic (F, Y only) residues are shown in red, green, and blue.

Table S2. Proteins identified by MS/MS after VAP-A and VAP-B pull-down. Proteins were precipitated by WT (Sample 1) and KD/MD mutant (Sample 2) VAP-A, and WT (Sample 3) and KD/MD mutant (Sample 4) VAP-B and identified by MS/MS. For each protein, the UniProt ID, name, score, coverage, number of peptides, and PSM are indicated.

Table S3. Total number of fly homologs/genes tested in the genetic screen. The genetic cross used to generate progeny for screening is shown at the top of the table. To perform this screening, we have used *rdgB*^*9*^ recombined with Gal4 cassette under rhodopsin 1 (Rh1) promoter at the first chromosome (blue). This parental line was used to cross with each RNAi line expressing dsRNA against the specific fly gene (orange). Each fly gene is denoted with their specific CG number (www.flybase.org). Highlighted in red were those genotypes whose RNAi lines were not available. Highlighted in green were those genotypes where RNAi/genotype did not yield any flies after the cross.

### Strategy of a genetic screen

**Figure 2.**
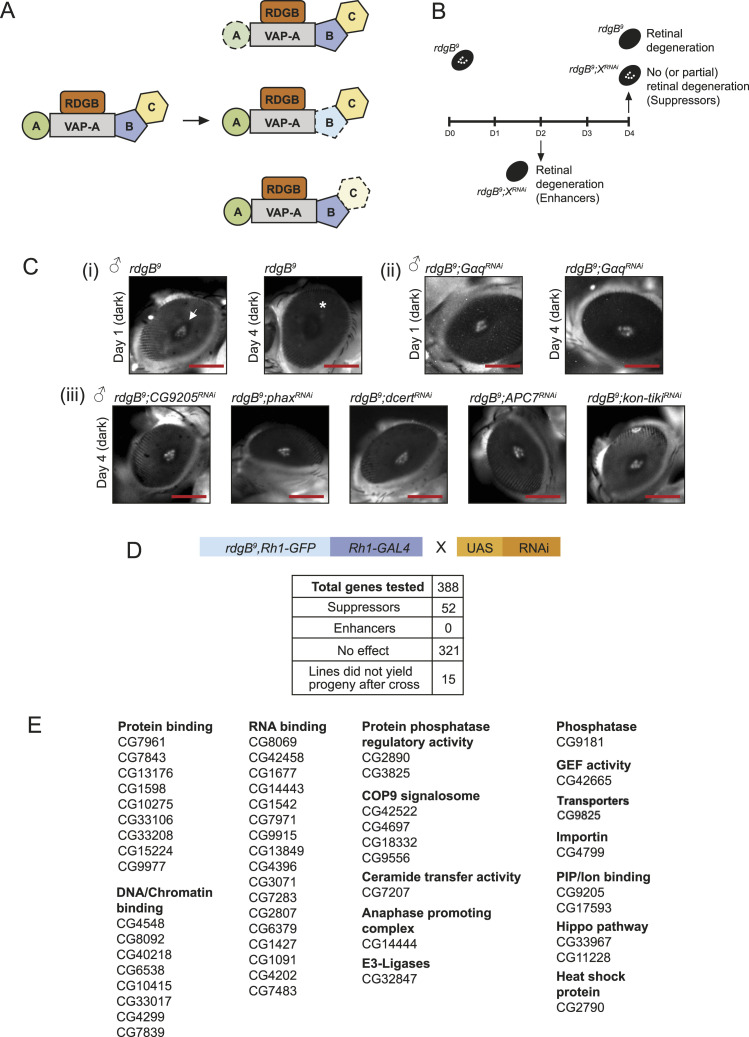
Strategy of the genetic screen and hits found. **(A)** Cartoon depicting classes of VAP interactors used in the present genetic screen. Three classes of genetic interactors of *rdgB* are shown based on the likely molecular mechanism: loss of A, a direct physical interactor of VAP-A; loss of B, a direct interactor of VAP-A that also interacts with C, a protein required for RDGB function; and loss of C, a protein required for *rdgB* function but only interacts with VAP-A via B. Depletion of a specific VAP interactor is depicted with a dotted line. Fly homologs were filtered using DIOPT in FlyBase (http://flybase.org/). **(B)** Genetic scheme used to find either enhancers or suppressors of the retinal degeneration phenotype of *rdgB*^*9*^. **(C)** Pseudopupil imaging: (i) *rdgB*^*9*^ showed retinal degeneration by day 4 in dark when checked via deep pseudopupil imaging (depicted by *). (ii) The degeneration was partially suppressed when levels of G_αq_ were down-regulated in *rdgB*^*9*^ on day 4. (iii) Selected hits that showed suppression of retinal degeneration in *rdgB*^*9*^ on day 4 (scale bar 225 μm). **(D)** Table showing the full list of genes used in the screen and the number of suppressor genes identified. **(E)** Positive hits (suppressor genes) are divided into different categories depending on their cellular functions. n = 5 flies/RNAi line.

To identify in vivo regulators of RDGB function at ER-PM contact sites, we used a hypomorphic allele *rdgB*^*9*^ (Vihtelic et al, 1991). *rdgB*^*9*^ expresses a small amount of residual RDGB protein that provides some function in contrast to the protein null allele *rdgB*^*2*^. The FFAT motif of RDGB interacts with the ER-resident membrane protein dVAP-A to provide both localization and function to RDGB (Yadav et al, 2018). FFAT motifs are found in many proteins of varied biological functions and serve to localize them to ER contact sites through a protein–protein interaction with VAP (Murphy & Levine, 2016). We reasoned that if several proteins with an FFAT motif bind to VAP at the ER-PM interface, the lipid transfer function of RDGB could be modulated by their presence at the ER-PM MCS (Fig 2A). Such proteins relevant to RDGB function could be identified by testing their ability to modify the phenotype of the *rdgB* mutant.

*rdgB*^*9*^ shows retinal degeneration that is enhanced when flies are grown under illumination (Harris & Stark, 1977; Stark et al, 1983). Under illumination, *rdgB*^*9*^ flies show severe retinal degeneration by 2 d post-eclosion, making it difficult to score for modulation of this phenotype by other gene products. To overcome this problem, we reared *rdgB*^*9*^ flies without illumination, a condition under which the retinal degeneration still occurs but at a slower rate; in dark-reared *rdgB*^*9*^ flies, it takes 2 d for the retinal degeneration to set in, and by day 4, complete retinal degeneration was seen (Fig 2B). Retinal degeneration was scored by visualizing the deep pseudopupil (DPP) under a fluorescence stereomicroscope (Georgiev et al, 2005). To visualize fluorescent pseudopupil, a protein fusion of rhodopsin 1 (Rh1) was tagged with GFP, expressed under its own promoter, and recombined in *rdgB*^*9*^. Under these conditions, *rdgB*^*9*^ shows a clear fluorescent DPP on day 1 that is lost by day 4 with the progression of retinal degeneration (Fig 2Ci).

To identify molecules regulating RDGB function, we depleted their mRNA levels using transgenic RNAi from publicly available collections (Dietzl et al, 2007; Perkins et al, 2015); for 5 of 393 fly genes, there was no RNAi line available from public resources (Table S3). The eye-specific Rh1 promoter was used to restrict GAL4 expression and thus gene depletion, in space to the outer six photoreceptors and in time to post–70-h pupal development (Yadav et al, 2015). To validate the genetic screen, G_αq_ was down-regulated in the *rdgB*^*9*^ flies and the pseudopupil was scored after days 2 and 4. Knocking down G_αq_ in *rdgB*^*9*^ flies under Rh1 promoter showed partial suppression of retinal degeneration, and hence, pseudopupil presence after day 4 in dark suggested the efficacy of the screening method (Fig 2Cii).

Using this strategy, we depleted each of the 388 VAP-interacting proteins via RNAi in the *rdgB*^*9*^-sensitized background (Fig 2Ciii, Table S3). The screen was performed such that the phenotype arising from off-targets could be minimized. We first used a single RNAi line per gene of interest for the pseudopupil analysis, and once a positive phenotype was scored, the assay was repeated with a second independent RNAi line for the same gene. Only those genes were finally tabulated where two independent lines per gene showed a positive phenotype. To assay the enhancement of retinal degeneration, fly eyes were visualized on day 2, whereas for suppression, fly eyes were checked on day 4. Any suppressor that showed complete recovery of DPP was scored as a full rescue, whereas others were designated as partial suppressors.

Of 388 genes, knockdown of 52 (two independent RNAi lines per gene) in *rdgB*^*9*^ showed suppression of retinal degeneration (Fig 2D, Table 1) (Table S4); we designated these as *su(rdgB)*. In this study, we did not identify any candidate that showed enhancement of degeneration when depleted in *rdgB*^*9*^. Moreover, 15 genes where only a single RNAi line was available, when tested, did not result in adult progeny (larval death/pupae formed but no fly emerged). Based on their Gene Ontology tags, the 52 *su(rdgB)* could be classified into several categories (Fig 2E). Of these, the largest number of suppressors was from the class of RNA binding and DNA/chromatin binding proteins. Examples of candidates with strong suppression phenotypes are the pleckstrin homology (PH) domain–containing protein CG9205, phosphorylated adaptor for RNA export (PHAX), ceramide transfer protein (Cert), anaphase-promoting complex 7 protein (APC7), and laminin G domain–containing protein Kon-tiki (Fig 2Ciii). These findings indicate that the mechanisms underlying retinal degeneration in *rdgB*^*9*^ likely involve diverse sub-cellular processes.

**Table 1.** List of *rdgB* interactors.

Total genetic interactors	Primary accession number (UniProt)	First RNAi line ID	Suppression	Second RNAi line ID	Suppression	Function	Human ortholog	Human primary accession number (UniProt)	Sequence identity with fly homologs (%)	Associated phenotypes	OMIM number
*CG8069*	A1Z7P3	100778/KK	++	28189/GD	++	Phosphorylated adaptor for RNA export	PHAX	Q9H814	29.1		604924
*CG4548*	Q9GQN5	101568/KK	++	10618/GD	++	XNP/adenosinetriphosphatase	ATRX	P46100	40.56	Alpha-thalassaemia/mental retardation syndrome	300032
*CG7961*	Q9W0B8	35305/GD	++	35306/GD	+	Coat protein (coatomer) α	COP-A	P53621	71.64	Autoimmune interstitial lung, joint, and kidney disease	601924
*CG7843*	Q9V9K7	106344/KK	+	22574/GD	+	Arsenic resistance protein 2	SRRT (Isoform 5)	Q9BXP5	46.58		614469
*CG42665*	Q9VVC6	105885/KK	+	101144/KK	+	Ephexin	ARHGEF5	Q9BXP5	31.12	Breast cancer	600888
*CG8092*	A0A0B4KER0	28196/GD	+	TRiP 25971	+	Relative of WOC	POGZ (Isoform 5)	Q7Z3K3	21.1	White–Sutton syndrome	614787
*CG42458*	Q7KU81	106608/KK	++	108072/KK	++	UN, mRNA binding	HNRNPC (Isoform 4)	P07910	29.73		164020
*CG42522*	Q7KTH8	TRiP 33370	++	No 2nd RNAi available		COP9 signalosome subunit 8	COPS8 (Isoform 2)	Q99627	24.73		616011
*CG1677*	Q9W3R9	109697/KK	++	50195/GD	+	UN, predicted to be involved in mRNA splicing, via spliceosome	ZC3H18 (Isoform 2)	Q9BXP5	32.12		Not applicable
*CG14443*	Q9W3Y5	105254/KK	++	17618/GD	++	UN, RNA helicase	DDX21	Q9NR30	22.99		606357
*CG1542*	Q9V9Z9	104575/KK	++	39976/GD	++	UN, predicted to be involved in rRNA processing and ribosomal large subunit biogenesis	EBNA1BP2	Q99848	42.61		614443
*CG9825*	Q9W1Z1	105868/KK	++	1712/GD	++	UN, solute carrier family 17 (SLC17) member	SLC17A7	Q13428	16.41		605208
*CG9205*	Q9W0K9	107612/KK	+	29079/GD	++	UN, oxysterol binding protein; PH domain	OSBPL11	Q9BXB4	37.5		606739
*CG7971*	A8JNI2	101384/KK	++	34262/GD	+	UN, predicted to be involved in RNA splicing	SRRM2	Q9UQ35	29.45	Intellectual developmental disorder, autosomal dominant 72	606032
*CG4799*	P52295	102627/KK	+	32466/GD	++	Pendulin	KPNA6	P52292	50.58		610563
*CG9915*	A8JV07	103731/KK	++	No 2nd RNAi available		UN, predicted to be involved in poly(A)+ mRNA export from the nucleus	IWS1 (Isoform 2)	Q96ST2	32.63		Not applicable
*CG13849*	Q95WY3	103738/KK	+	51775/GD	+	Nop56	NOP56	O00567	62.73	Spinocerebellar ataxia 36	614154
*CG9181*	Q9W0G1	108888/KK	++	37436/GD	++	Protein tyrosine phosphatase 61F	PTPN12	Q05209	26.51	Colon cancer, somatic	600079
*CG4396*	Q9VYI0	101508/KK	+	48891/GD	+	found in neurons	ELAVL1	Q15717	62.31		603466
*CG33967*	Q9VFG8	106507/KK	++	100765/KK	++	KIBRA	WWC1	Q8IX03	37.1	Memory, enhanced, QTL	610533
*CG13176*	Q7JW27	39769/GD	++	24642/GD	++	Washout	WASH6P	Q9NQA3	30.32		Not applicable
*CG3071*	Q9W4Z9	107206/KK	++	29589/GD	+	UN, predicted to have snoRNA binding activity	UTP15	Q8TED0	37.35		616194
*CG1598*	Q7JWD3	110555/KK	+	32391/GD	++	Unnamed/adenosinetriphosphatase	GET3	O43681	68.07	Cardiomyopathy, dilated, 2H	601913
*CG40218*	Q8SXI2	102960/KK	++	No 2nd RNAi available		Yeti	CFDP1	Q9UEE9	31.93		608108
*CG4697*	Q9VJR9	34308/GD	++	34307/GD	++	COP9 signalosome subunit 1a	GPS1	Q13098	37.11		601934
*CG14444*	Q9W3Y6	110729/KK	++	17622/GD	++	Anaphase-promoting complex subunit 7	ANAPC7 (Isoform 2)	Q9UJX3	25.36	Ferguson–Bonni neurodevelopmental syndrome	606949
*CG2890*	Q9W2U4	105399/KK	++	25445/GD	++	Protein phosphatase 4 regulatory subunit 2–related protein	PPP4R2 (Isoform 3)	Q9NY27	31.66		613822
*CG7283*	Q9VTP4	109345/KK	++	23459/GD	++	Ribosomal protein L10Ab	RPL10A	P62906	76.96		615660
*CG2807*	Q9VPR5	110091/KK	+	25162/GD	++	Splicing factor 3b subunit 1	SF3B1	O75533	79.95	Myelodysplastic syndrome, somatic	605590
*CG6538*	P41900	110569/KK	+	12602/GD	+	Transcription factor TFIIFβ	GTF2F2	P13984	50.83		189969
*CG18332*	Q8SYG2	101516/KK	+	12821/GD	++	COP9 signalosome subunit 3	COPS3	Q9UNS2	52.84		604665
*CG6379*	Q9W4N2	103723/KK	+	29611/GD	++	Unnamed/methyltransferase cap1	CMTR1	Q8N1G2	38.35		616189
*CG1427*	Q9VNE3	105727/KK	+	17456/GD	++	Sec synthetase	SEPSECS (Isoform 3)	Q9HD40	46.43	Pontocerebellar hypoplasia type 2D	613009
*CG10275*	Q9VJ82	106680/KK	++	37283/GD	++	Kon-tiki	CSPG4	Q6UVK1	24.52		601172
*CG2790*	Q9W0X8	101619/KK	+	20903/GD	+	UN, the heat shock protein 40 (Hsp40) family of co-chaperones	DNAJC21	Q8N7S2	30.95	Bone marrow failure syndrome 3	617048
*CG10415*	O96880	100572/KK	+	12592/GD	+	Transcription factor IIEα	GTF2E1	P29083	46.23		189962
*CG11228*	Q8T0S6	104169/KK	++	7823/GD	+	Hippo	STK3	Q13188	58.45		605030
*CG1091*	Q9VI58	107175/KK	+	16088/GD	++	Tailor, RNA uridylyltransferase	TUT1	Q9H6E5	23.19		610641
*CG33106*	Q9VCA8	103411/KK	++	33394/GD	+	mask, multiple ankyrin repeats, single KH domain	ANKRD17 (Isoform 6)	O75179	47.77	Chopra–Amiel–Gordon syndrome	615929
*CG33208*	Q86BA1	105837/KK	+	25371/GD	+	MICAL, molecule interacting with CasL	MICAL3	Q7RTP6	33.76		608882
*CG15224*	P08182	106845/KK	+	32377/GD	+	Casein kinase II β subunit	CSNK2B	P67870	87.91	Poirier–Bienvenu neurodevelopmental syndrome	115441
*CG17593*	Q9VQR9	106469/KK	++	13029/GD	++	UN, orthologous to human CCDC47 (coiled-coil domain containing 47)	CCDC47	Q96A33	43.52	Trichohepatoneurodevelopmental syndrome	618260
*CG33017*	A1ZAC8	103968/KK	+	40022/GD	+	UN, the MADF-BESS domain transcription regulators	GPATCH8 (Isoform 2)	Q9UKJ3	21.17		614396
*CG4299*	P53997	108987/KK	+	TRiP 77433	+	Set, encodes a subunit of the inhibitor of the histone acetyltransferase (INHAT) complex	SET	Q01105	58.17	Intellectual developmental disorder, autosomal dominant 58	600960
*CG7207*	Q9Y128	103563/KK	++	27914/GD	+	Ceramide transfer protein	CERT	Q9Y5P4	44.31	Intellectual developmental disorder, autosomal dominant 34	604677
*CG4202*	Q9I7W5	103352/KK	++	49946/GD	+	Something about silencing 10	UTP3	Q9NQZ2	37.2		611614
*CG9977*	Q9VZX9	106749/KK	++	36193/GD	++	Adenosylhomocysteinase-like 1	AHCYL1	O43865	72.87		607826
*CG32847*	Q8IQM1	104294/KK	++	48423/GD	++	UN, contains the RING (Really Interesting New Gene) finger domain	TRIM26	Q12899	21.25		600830
*CG7839*	Q9VTE6	105979/KK	+	12691/GD	+	UN, orthologous to human CEBPZ (CCAAT/enhancer binding protein zeta).	CEBPZ	Q03701	29.87		612828
*CG7483*	Q9VHS8	108580/KK	++	TRiP 32444	+	eIF4AIII, ATP-dependent RNA helicase	EIF4A3	P38919	86.97	Robin sequence with cleft mandible and limb anomalies	608546
*CG9556*	Q94899	48044/GD	++	TRiP 28908	++	Alien	COPS2	P61201	83.97		604508
*CG3825*	Q9W1E4	107545/KK	+	TRiP 33011	+	Protein phosphatase 1 regulatory subunit 15	PPP1R15B	Q5SWA1	22.33	Microcephaly, short stature, and impaired glucose metabolism 2	613257

Summary of *Drosophila rdgB* genetic interactors identified in the screen. Gene name and/or CG number in FlyBase (www.flybase.org) and UniProt (https://www.uniprot.org/) accession number along with their GO functional annotation. For each gene, the ID of RNAi lines from the VDRC or TRiP library used is shown. Phenotypes scored after depletion of each gene are represented under the “Suppression” column; “++” denotes definite suppression, whereas “+” denotes partial suppression. The human ortholog of each *rdgB* interactor is identified. Known phenotypes associated with each human homolog are denoted along with the Online Mendelian Inheritance in Man (OMIM) identifier number.

Table S4. Table showing each of the 52 *su(rdgB)* with their potential FFAT motifs and their respective human homolog. For each human and fly protein, the UniProt ID, and the two best conventional and Phospho-FFAT scores are indicated. The position and the sequence of potential FFAT sequences are indicated. FFAT scores are colour-coded with a scale from orange to blue (dark to light orange: 0–3; light to dark blue: 3.5->5). Acidic, phosphorylatable (S, T only), and aromatic (F, Y only) residues are shown in red, green, and blue.

### Identification of suppressors specific to *rdgB*^*9*^

In principle, depletion of a gene product can suppress retinal degeneration in *rdgB*^*9*^ by one of the two mechanisms: (i) by altering the underlying biochemical abnormality resulting from loss of RDGB function, that is, the trigger; and (ii) by down-regulating downstream sub-cellular processes that are part of the degenerative process, that is, the effectors. Genes in the first category, that is, the trigger mechanism, might be expected to suppress only the degeneration of *rdgB*^*9*^ and no other retinal degeneration, whereas genes that are effectors of retinal degeneration might be expected to suppress multiple retinal degeneration mutants.

**Figure 3.**
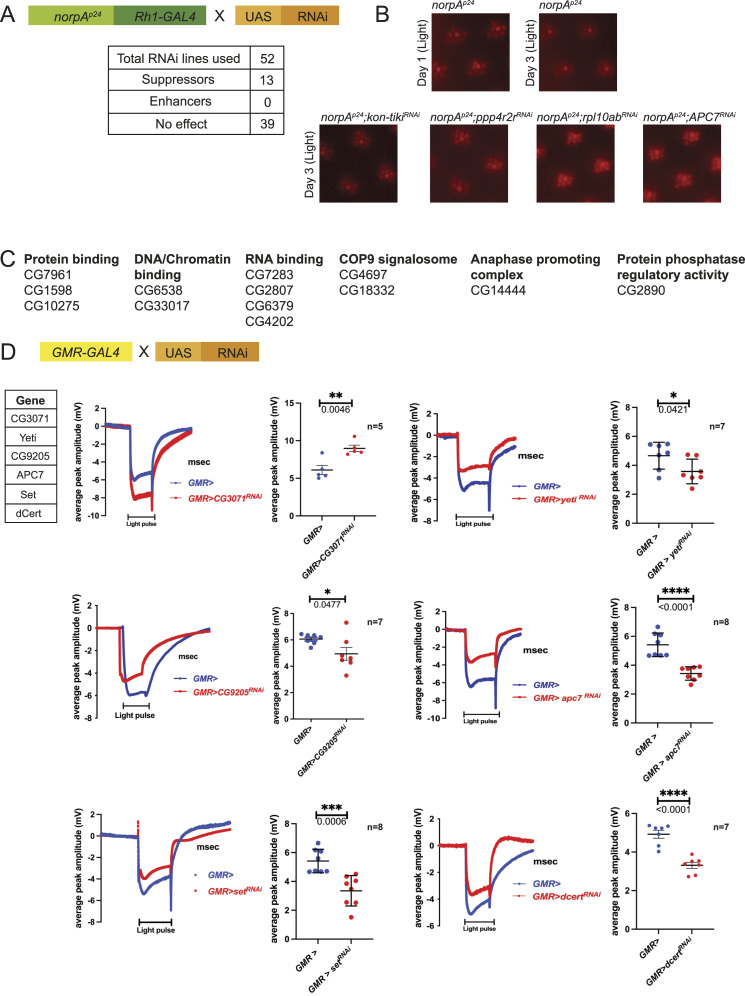
Genetic screen using *norpA*^*p24*^. **(A)** Scheme used to test for genetic interaction of each of the 52 *su(rdgB)* with *norpA*^*p24*^ under illumination conditions (constant light 2000 Lux). **(B)** *norpA*^*p24*^ flies degenerate by day 3 under light conditions, and examples of *su(RDGB)* candidates that suppressed the *norpA*^*p24*^ retinal degeneration phenotype. n = 5 flies/RNAi line. **(C)** Complete list of 13 genes with their cellular functions that suppressed the *norpA*^*p24*^ phenotype. ERG screen. **(D)** Of 52 candidates, five *su(RDGB)* showed reduced (CG9205, Yeti, Apc7, Set, and dCert) and one (CG3071) showed higher ERG phenotype (traces and quantification shown) when down-regulated in an otherwise WT background. The number of flies used for the experimental set is mentioned along with the quantification. Scatter plots with the mean ± SEM are shown. Statistical tests: unpaired *t* test.

To distinguish these two categories of genes, we tested each of the 52 *su(rdgB)* for their ability to block retinal degeneration in *norpA*^*p24*^ (Fig 3A and Table S5). *norpA* encodes for the PLC and catalyses the hydrolysis of PI(4,5)P_2_ to DAG and IP_3_. *norpA*^*p24*^ is a strong hypomorph and shows light-dependent retinal degeneration (Pearn et al, 1996). Of the 52 *su(rdgB)*, 13 genes partially suppressed light-dependent retinal degeneration in *norpA*^*p24*^, suggesting that they likely participate in the process of retinal degeneration (Fig 3B and C). Most genes in this category belong to the class of RNA binding/processing and DNA/chromatin binding (Fig 3C). The remaining 39 genes therefore likely represent unique suppressors of *rdgB*^*9*^ and therefore may participate specifically in the trigger mechanism.

Table S5. Table showing each of the 52 *su(rdgB)* with their respective RNAi line tested for suppression of retinal degeneration in *norpA*^*p24*^. The genetic cross used to generate progeny for screening is shown at the top of the table. To perform this screening, we have used *norpA*^*p24*^ recombined with Gal4 cassette under rhodopsin 1 (Rh1) promoter at the first chromosome (green). This parental line was used to cross with each RNAi line expressing dsRNA against the specific fly gene (orange). Each fly gene is denoted with their specific CG number (www.flybase.org). KK/GD with a specific identifier number denotes the RNAi library generated by Vienna Drosophila Resource Centre. Any suppression of retinal degeneration in *norpA*^*p24*^ by down-regulating the specific *su(rdgB) under* the Rh1 promoter is denoted by “Yes.”

### ERG screen to identify *su(rdgB)* that may regulate phototransduction

**Figure S1.**
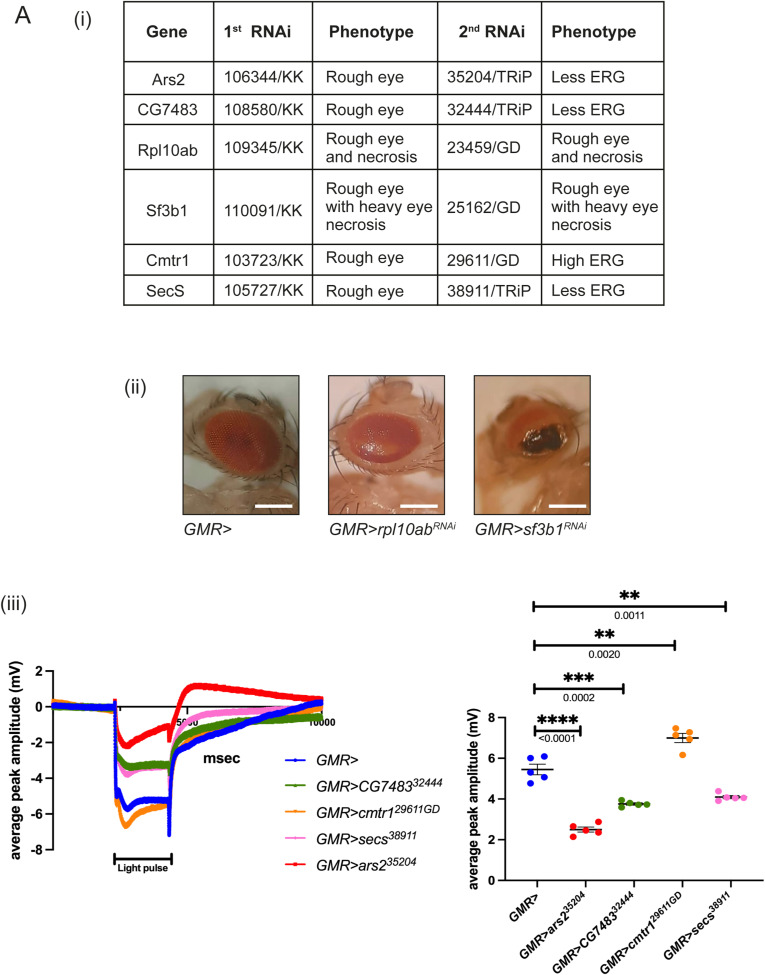
Six *su(rdgB)* affecting either eye development or physiology. **(A)** Second category of *su(rdgB)* was variable in either showing a rough eye phenotype in the first RNAi line and ERG defects in the second independent RNAi line. (ii) *rpl10Ab* and *sf3b1* are the only candidates that consistently showed a rough eye phenotype in two independent RNAi lines (scale bar 225 μm). (iii) ERG traces and quantifications of the rest of the *su(rdgB)* with their respective RNAi line mentioned. The number of flies used for the experimental set is mentioned along with the quantification. Scatter plots with the mean + SEM are shown. Statistical tests: unpaired *t* test.

A direct test of the role of a candidate in regulating phototransduction will be its ability, when depleted in an otherwise WT fly, to alter the electrical response to light. This can be monitored using ERG that are extracellular recordings that measure the electrical signal from the eye in response to a light stimulus (Vilinsky & Johnson, 2012). Any deviation of the ERG amplitude when compared to that from a WT fly will imply that the interactor likely functions in the process of phototransduction. We down-regulated each *su(rdgB)* using the eye-specific promoter, GMR-GAL4, in an otherwise WT background and measured ERG amplitudes. Of 52 *su(rdgB)*, GMR-driven knockdown (in both of two independent RNAi lines) of five candidates (CG9205, Yeti, APC7, Set, and Cert) showed a lower ERG amplitude and of one candidate (CG3071) a higher ERG amplitude compared with control flies (Fig 3D and Table S6). In the case of six additional *su(rdgB)*, depletion with GMR-GAL4 resulted in a rough eye phenotype with the first RNAi line (Fig S1Ai). When a second independent RNAi line was used, four (Ars2, CG7483, Cmtr1, and Secs) of six candidates showed lower ERG amplitude (Fig S1Aiii). A rough eye phenotype after knocking down Rpl10ab and Sf3b1 with multiple RNAi lines points towards involvement of these genes in the eye development (Fig S1Aii).

Table S6. Table showing each of the 52 *su(rdgB)* with their respective RNAi line tested for ERG/developmental phenotype when tested in an otherwise WT background under GMR-Gal4. Each gene is denoted by their specific CG number. KK/GD denotes the RNAi library generated by Vienna Drosophila Resource Centre, whereas TRiP lines denote the RNAi library procured from Bloomington Drosophila Resource Centre. Where available, KO lines were used. Phenotypes scored are denoted under the “ERG” column.

### The spatial and temporal profile of dCert and CG9205 down-regulation results in contrasting impact on *rdgB*^*9*^ phenotypes

**Figure 4.**
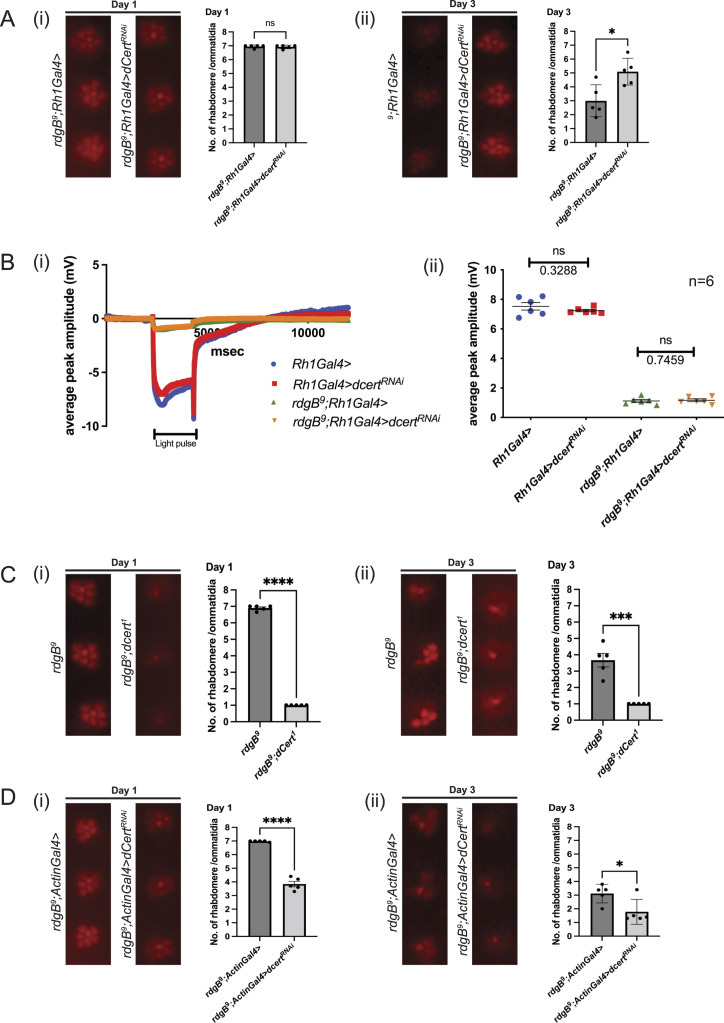
Spatial and temporal down-regulation of dCert in *rdgB*^*9*^. **(A)** Suppression of retinal degeneration when dCert RNAi line (35579/TRiP, BDRC) was expressed using Rh1 promoter. After eclosion, flies were kept in the dark and assayed on either day 1 or 3: (i) on day 1, there was no appreciable difference in two genotypes and rhabdomeres were intact; and (ii) on day 3, down-regulation of dCert in *rdgB*^*9*^ suppressed the retinal degeneration observed in *rdgB*^*9*^ control. **(B)** When subjected to ERG analysis, down-regulation of dCert using Rh1-GAL4 in the background of *rdgB*^*9*^ did not suppress the ERG phenotype: (i) ERG trace and (ii) quantification. n = 6 flies. Scatter plots with the mean ± SEM are shown. Statistical tests: unpaired *t* test. **(C)** Double mutant of *rdgB*^*9*^;*dcert*^*1*^ showed enhancement of retinal degeneration: (i, ii) by day 1 alone, double mutant has severely enhanced retinal degeneration phenotype when compared to *rdgB*^*9*^. **(D)** Enhancement of retinal degeneration when dCert (35579/TRiP, BDRC) was down-regulated with a whole-body Actin-Gal4 promoter in the *rdgB*^*9*^ background: (i) on day 1, rhabdomere loss is significant in the experimental files compared with control that worsens by day 3 and phenocopies the retinal degeneration present in the double mutant. For optical neutralization experiments, scoring was done by quantifying 10 ommatidia/fly head, n = 5 fly heads.

**Figure 5.**
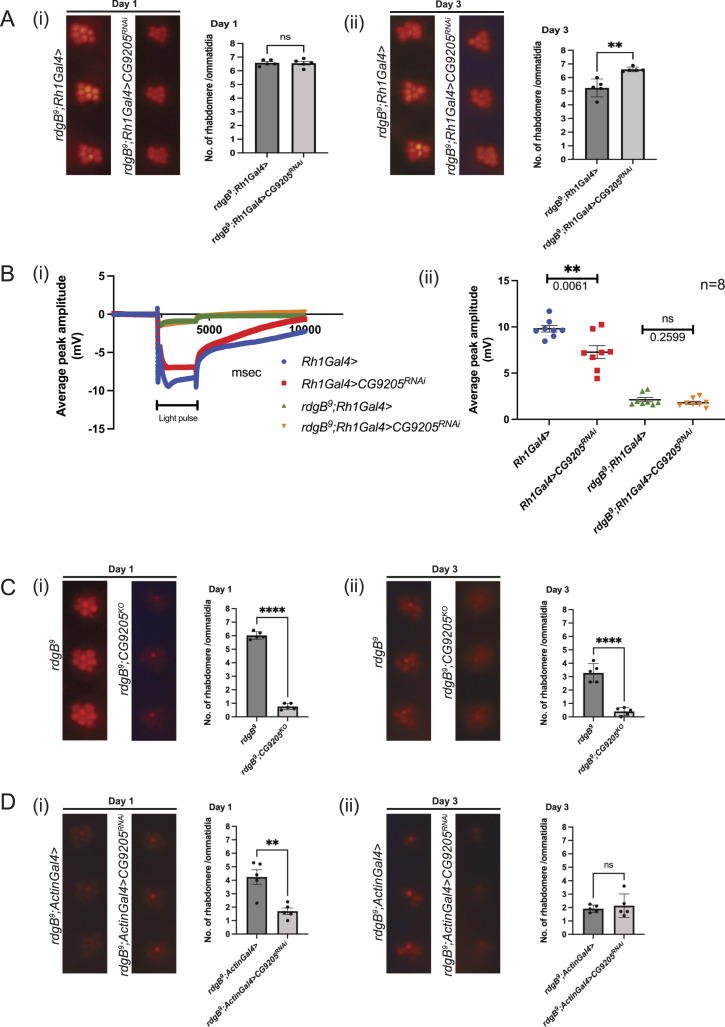
Spatial and temporal down-regulation of CG9205 in *rdgB*^*9*^. **(A)** Suppression of retinal degeneration when CG9205 RNAi line (29079/GD, VDRC) was expressed using Rh1 promoter. After eclosion, flies were kept in the dark and assayed on either day 1 or 3: (i) on day 1, there was no appreciable difference in two genotypes and rhabdomeres were intact; and (ii) on day 3, down-regulation of CG9205 in *rdgB*^*9*^ suppressed the retinal degeneration observed in *rdgB*^*9*^ control. **(B)** When subjected to ERG analysis, down-regulation of CG9205 using Rh1-GAL4 in the background of *rdgB*^*9*^ did not suppress the ERG phenotype, whereas down-regulation of CG9205 using Rh1-GAL4 in an otherwise WT background shows reduced ERG amplitude: (i) ERG trace and (ii) quantification. n = 8 flies. scatter plots with the mean ± SEM are shown. Statistical tests: unpaired *t* test. **(C)** Double mutant of *rdgB*^*9*^;*CG9205*^*KO*^ showed enhancement of retinal degeneration: (i, ii) by day 1 alone, double mutant has severely enhanced retinal degeneration phenotype when compared to *rdgB*^*9*^. **(D)** Enhancement of retinal degeneration when CG9205 (29079/GD, VDRC) was down-regulated with a whole-body Actin-Gal4 promoter in the *rdgB*^*9*^ background: (i) on day 1, rhabdomere loss is significant in the experimental files compared with control that remains the same on day 3 and phenocopies the retinal degeneration present in the double mutant. For optical neutralization experiments, scoring was done by quantifying 10 ommatidia/fly head, n = 5 fly heads.

To confirm the findings of our RNAi depletion studies, we sought to study the impact of germline mutations in candidates identified in the RNAi screen on *rdgB*^*9*^. Of the six genes identified as specific *rdgB* interactors, *CG9205*, *yeti*, *APC7*, *set*, *dcert*, and *CG3071*, there were no mutants available in two of them (*set* and *CG3071*). Mutants in *yeti* are homozygous lethal, making it difficult to work on it in this setting. A viable mutant in *APC7* is available, but the encoded protein has no obvious membrane interaction domains. However, as dCert and CG9205 have membrane-interacting domains, we chose to focus on these two candidates for the proof-of-principle analysis. As previously noted, down-regulation of dCert in *rdgB*^*9*^ caused suppression of retinal degeneration when the RNAi construct was expressed using the Rh1 promoter (Fig 4Ai and ii), although the suppression of retinal degeneration was not sufficient to rescue the ERG phenotype of *rdgB*^*9*^ (Fig 4Bi and ii). We retested this genetic interaction using a germline mutant allele of *dcert* (*dcert*^*1*^) (Rao et al, 2007). Surprisingly, the double mutant *rdgB*^*9*^*;dcert*^*1*^ showed enhancement of retinal degeneration compared with *rdgB*^*9*^ (Fig 4Ci and ii). We confirmed these findings using the same dCert RNAi line used in the screen (expressed using Rh1 Gal4) but this time with the whole-body expression of the RNAi using Actin-GAL4 that expresses throughout development beginning with embryogenesis. In *rdgB*^*9*^*;actin>dcert*^*RNAi*^, we found enhancement of retinal degeneration such that by day 3, all photoreceptors except R7 were completely degenerated (Fig 4Di and ii), thus recapitulating the observations seen with *rdgB*^*9*^*;dcert*^*1*^. In a similar fashion, down-regulating CG9205, which encodes a PH domain–containing protein, under the Rh1 promoter resulted in the suppression of retinal degeneration in *rdgB*^*9*^ (Fig 5Ai and ii). When subjected to ERG, the suppression of retinal degeneration did not result in ERG rescue, whereas in an otherwise WT background, reduction in CG9205 levels under Rh1 showed a reduced ERG amplitude (Fig 5Bi and ii). In contrast, when a germline CG9205 CRISPR knockout (*CG9205*^*KO*^) was combined with *rdgB*^*9*^, there was enhanced photoreceptor degeneration (Fig 5Ci and ii). This result was corroborated using the whole-body promoter, Actin-Gal4, to reduce CG9205 levels in the *rdgB*^*9*^ background where retinal degeneration was enhanced (Fig 5Di and ii). These findings suggest that dCert and CG9205 depletion more broadly in the fly across both space and time domains may have distinctive effects compared with a more restricted expression in post-mitotic adult photoreceptors using Rh1 GAL4. This finding also suggests multiple modes of action for dCert and CG9205 in *Drosophila* photoreceptors during distinct phases of photoreceptor development.

## Discussion

Neurodegeneration is a complex process involving multiple layers of cellular and molecular events leading to the phenotype observed in vivo. Regardless of the part of the nervous system that is affected, be it the central or peripheral, conceptually, the processes leading to any neurodegeneration can be classified into two groups: (i) trigger steps—that is, those initial molecular or biochemical changes that initiate the process of degeneration; and (ii) effector steps—that is, those steps that are subsequently part of the process that leads to loss of neuronal structure and consequently function. Identifying the molecular processes involved in each of these steps is critical for developing strategies to manage neurodegenerative disorders. The *Drosophila* eye has been used in several settings for modelling neurodegeneration (Bonini & Fortini, 2003) such as those caused by repeat disorders such as Huntington’s disease and various ataxias, Alzheimer’s disease, and primary degenerative disorders of the human retina (Xiong & Bellen, 2013). In the present study, we performed a genetic analysis to uncover the mechanisms of retinal degeneration underlying mutants in *rdgB*, which encodes a Class II PITP. Mutations in Class I PITP (PITPα) in mice result in a neurodegeneration phenotype (Hamilton et al, 1997), and recently, human patients carrying mutations in VPS13 have been reported with neurodegenerative disorders (Ugur et al, 2020). Thus, the findings of our screen will inform on mechanisms of neurodegeneration.

**Figure S2.**
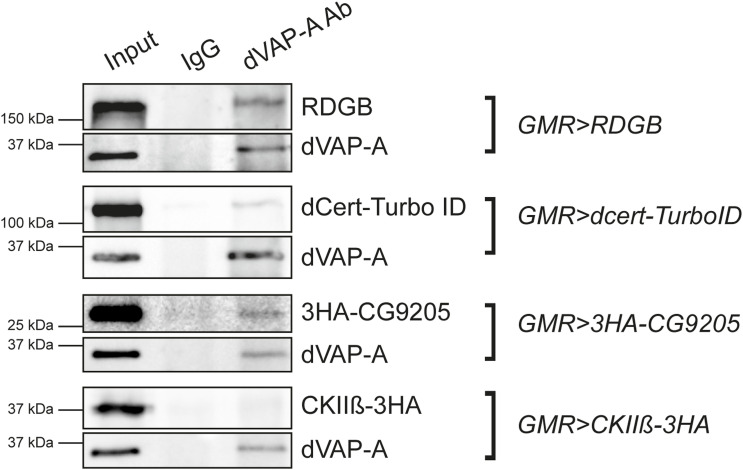
Representative immunoblots showing co-immunoprecipitation of RDGB (used as a control) and dCert and CG9205 after pulling with dVAP-A antibody in fly heads. UAS constructs of RDGB, dCert::TurboID, and 3HA::CG9205 were expressed under GMR promoter. The experiment was repeated twice.

The ER-resident protein VAP that has been linked to neurodegenerative diseases (Nishimura et al, 2004) is known to interact with multiple cellular proteins via the binding of its MSP domain with the FFAT motif of other proteins (Murphy & Levine, 2016). In this study, we performed an immunoprecipitation analysis that exploits this FFAT-VAP interaction and were able to identify 401 proteins enriched in an IP-MS experiment. Surprisingly, only 136 of these proteins had an identifiable FFAT or Phospho-FFAT motif. This is despite the list of 401 proteins being curated as those enriched in an IP-MS with WT VAP but not a non-FFAT binding version of this protein. This implies that 265 proteins (66%) of the total proteins identified in the IP-MS experiment do not interact directly with VAP but do so indirectly presumably via other proteins (Fig 2A). This suggests that VAP-interacting proteins in cells include both categories (a smaller direct FFAT-VAP–meditated set and a larger indirect interactor set) and both groups may influence molecular processes in the vicinity of VAP. How significant are the interactions of VAP with these non–FFAT-containing proteins identified in the IP-MS proteomics experiment? When tested experimentally, we found that one such protein CG9205 could interact with VAP in an immunoprecipitation experiment from *Drosophila* head extracts (Fig S2). In contrast, another such non–FFAT-containing interactor casein kinase II β did not interact with VAP under similar conditions (data not shown). Interestingly, a previous large-scale experimental study of cellular protein–protein interactions that described a VAP interactome through mass spectrometry–based methods also noted non–FFAT-containing proteins such as LSG1 identified as interactors of VAP in mammalian cells (Huttlin et al, 2015). Of 38 VAP interactors identified in the BioPlex study, only 12 have identifiable FFAT motifs. These findings highlight the possibility that VAP-interacting proteins identified using proteomics screens may also include both direct and indirect interacting groups. It also suggests that although bioinformatics-based identification of FFAT motifs in sequenced genomes from other systems will continue to be a useful means of identifying VAP-interacting proteins, experimental methods such as IP-MS will add to the identification of the larger group of VAP-interacting proteins, many of whom may not have FFAT motifs.

To understand the cellular and molecular processes underlying retinal degeneration in *rdgB^9^*, we depleted selected molecules using RNAi and scored for suppression of retinal degeneration. The candidates selected for screening were initially identified using an IP-MS proteomics screen for interactors of VAP-A and VAP-B in cultured mammalian cells; however, the functional significance of their interaction with VAP was not known. Although previous studies have identified many VAP-interacting proteins in mammalian cell culture models by protein interaction studies, the functional relevance of these for in vivo function and neurodegeneration remains unknown. Using our in vivo analysis, we were able to identify a subset (52 of 388) of these interactors in our proteomics screen that, when depleted, suppressed the retinal degeneration in *rdgB*^*9*^. This finding underscores the value of an in vivo genetic screen in evaluating the functional effect of candidates identified in vitro to understanding the mechanisms of neurodegeneration. The human homologs in 13 of the *su(rdgB)* genes have previously been linked to human neurodevelopmental or neurodegenerative disorders (Table 1), and a large proportion of the 52 *su(rdgB)* have human homologs that show high expression in the human brain (Table S7). Thus, the findings of this study could provide important insights into the mechanisms of human brain disorders.

Table S7. Levels of mRNA and protein of the identified genetic interactors of *rdgB* in the brain. The data for mRNA expression and protein expression have been obtained from the Human Protein Atlas database (https://www.proteinatlas.org/) for the human homologs of the 52 genes reported as genetic interactors of *rdgB*. For the mRNA expression, the consensus TPM values from HPA in the cerebral cortex and cerebellum (including HPA, GTEx, and Fathom data) have been mentioned in columns 4 and 5. The protein expression of the genes (mentioned as low, medium, or high in HPA) has been shown for the cerebral cortex and cerebellum in columns 2 and 3. HPA reports the protein expression in various cell types of the brain; however, the region of the cerebral cortex and cerebellum with the highest expression has been used to report the protein expression. The cells marked in “yellow” denote lack of data availability, whereas cells marked in “blue” denote low/no protein detected.

Because our primary screen for suppressors of *rdgB* would identify molecules involved in both the trigger and effector steps of the degeneration process, it is essential to classify the identified suppressers into these two categories. Because *rdgB* mutants are known to affect photoreceptor physiology before the onset of retinal degeneration (Yadav et al, 2015), we reasoned that suppressors that work at the level of the trigger might also affect the electrical response to light, the physiological output of the photoreceptor. By this rationale, we found that 6 of 52 suppressors when depleted in an otherwise WT background led to an altered electrical response to light; these suppressors are therefore likely to impact the processes by which RDGB functions in phototransduction. Examples of these include CG9205, Yeti, APC7, Set, Cert, and CG3071. Two of these genes *CG9205* (PH domain–containing) and *cert* (ceramide transfer protein) encode proteins with either ion binding or lipid transfer function, and their ability to act as *su(rdgB)* may indicate a role of previously unidentified lipids and lipid transfer at MCS in phototransduction. In contrast, Set (subunit of the INHT complex that regulates histone acetylation), Yeti (a chromatin-associated protein that interacts with the Tip60 chromatin remodelling complex), and CG3071 (snoRNA that positively regulates transcription by RNA polymerase 1) all likely exert their effect as *su(rdgB)* by modulating gene expression; some of the genes so regulated may impact phototransduction. A transcriptome analysis of *rdgB*^*9*^ photoreceptors may help identify the relevant genes and the manner in which they regulate phototransduction.

To identify molecular mechanisms that regulate the effector steps of the degeneration process, we determined which of the *su(rdgB)* could also suppress another retinal degeneration mutant, *norpA*^*p24*^. Such *su(rdgB)* will likely represent molecules that participate in common effector steps of retinal degeneration shared by these two mutants. The 13 genes so identified represent several different functional classes. Prominent among these classes are RNA binding and DNA/chromatin binding proteins. Overall, a large percentage of *su(rdgB)* identified in our screen were of the class of RNA processing (CG1677, CG1542, CG7971, Cmtr1, Srrm234, Nop56, CG3071, Rpl10, Ars2, CG42458, SecS, CG9915, Sf3b1), RNA editing (Tailor, Sas10), RNA export (Phax), and RNA helicases (CG14443, CG7483). Interestingly, a role of RNA binding proteins such as Ataxin-1 has been proposed in neuronal homeostasis and neurodegenerative processes and our finding may reflect a more general role of RNA binding/homeostasis in neurodegenerative processes (Prashad & Gopal, 2021). A further large group of *su(rdgB)* belong to those regulating transcription (XNP, Fne, Yeti, TFIIFβ, TFIIEα, CG33017, Set, CG7839), and Sf3b1, Cmtr1, Rpl10Ab, TFIIFβ, and Sas10 were among those candidates that in addition suppressed retinal degeneration in *norpA*^*p24*^. This finding suggests that regulated transcription may be important for maintaining neuronal homeostasis; this may be particularly significant because neurons are post-mitotic and transcriptional process and RNA turnover may collectively be key mechanisms for maintaining cellular homeostasis.

A third class of *su(rdgB)* were subunits of the COP9 signalosome (CSN1a, CSN2, CSN3, and CSN8 were identified in our screen). The COP9 signalosome acts as a signalling platform regulating cellular ubiquitylation status. The COP9 signalosome has been shown to play a key role in regulating *Drosophila* development through E3 ubiquitin ligases by deNEDDylation (Freilich et al, 1999). In addition, two E3 ubiquitin ligase family members were also identified in the genetic screen: (i) APC7, which is a subunit of anaphase-promoting complex/cyclosome that comprises seven other subunits and is required to modulate cyclin levels during cell cycle; and (ii) *CG32847*, an uncharacterized gene belonging to the “Other RING domain ubiquitin ligases” family of proteins. Ubiquitination could regulate the structure and function of proteins required for phototransduction; depletion of APC7 resulted in a reduction in the ERG amplitude supporting this mechanism. Alternatively, it is possible that ubiquitination-regulated protein turnover may be part of the process of retinal degeneration. Interestingly, a key role of ubiquitination has been described in the context of neurodegeneration (Schmidt et al, 2021).

Overall, our screen uncovers a role of multiple molecular processes regulated by VAP-interacting proteins that are required for maintaining lipid turnover and neuronal homeostasis in photoreceptors. It is important to note that our screen focused on VAP-interacting proteins, but there will also be non–VAP-dependent processes that also contribute to lipid and neuronal homeostasis in photoreceptors. Alternative genetic screens will be required to map their role in photoreceptor maintenance. Collectively, such studies will help advance our understanding of neurodegeneration in the context of LTP function.

## Materials and Methods

### Protein pull-down and mass spectrometry analysis

Recombinant protein expression in *E. coli* and purification using plasmids encoding the MSP domain of VAP-A (8–212; WT and KD/MD mutant) and VAP-B (1–210; WT and KD/MD mutant) were previously described (Di Mattia et al, 2020). For protein pull-down, the affinity resin was prepared by incubating 100 μg of recombinant protein with 20 μl of nickel beads (PureProteome Nickel Magnetic Beads; Merck) in pull-down buffer PDB (50 mM Tris–HCl, pH 7.4, 50 mM NaCl, 1 mM EDTA, 1% Triton X-100, 5 mM imidazole, cOmplete protease inhibitor cocktail [Roche], and PhosSTOP [Roche]). The beads were then washed three times with the same buffer. 8 × 10^8^ HeLa cells were washed with 5 ml of TBS and lysed with 1 ml of PDB. After a 10-min incubation on ice, the protein extract was separated from cell debris by centrifugation (10 min; 9,500*g*; 4°C). The protein extract was mixed with VAP-coupled nickel beads and incubated for 2 h at 4°C under constant agitation. The beads were then washed three times with PDB, and proteins were eluted with Laemmli buffer. Proteins were precipitated with trichloroacetic acid and digested with Lys-C (Wako) and trypsin (Promega). Peptides were then analysed using Ultimate 3000 nano-RSLC (Thermo Fisher Scientific) coupled in-line with Orbitrap ELITE (Thermo Fisher Scientific).

### SDS–PAGE, Western blot, and Coomassie blue staining

SDS–PAGE and Western blot analysis were performed as previously described (Alpy et al, 2005) using the following antibodies: rabbit anti-STARD3NL (1:1,000; pAbMENTHO-Ct-1545 [Alpy et al, 2001]), rabbit anti-ORP1 (1:1,000; ab131165; Abcam), and mouse anti-actin (1:5,000; A1978; Merck). Coomassie blue staining was performed with PageBlue Protein Staining Solution (Thermo Fisher Scientific).

### In silico identification of potential conventional and Phospho-FFAT motifs

The FFAT scoring algorithm used for Phospho-FFAT identification is based on the position weight matrix (Di Mattia et al, 2020). For conventional FFAT sequences, the Phospho-FFAT matrix described in Di Mattia et al was modified in position 4 to assign a score of 4 to S and T, and a score of 0 to D and E. These algorithms assign conventional and Phospho-FFAT scores to protein sequences. They are based on 19 continuous residues: six residues upstream, seven residues forming the core, and six residues downstream. An ideal sequence scores zero.

### Fly culture and stocks

Flies (*Drosophila melanogaster*) were reared on standard cornmeal, dextrose, yeast medium at 25°C and 50% relative humidity in a constant-temperature laboratory incubator. There was no internal illumination within the incubator, and flies were subject to brief pulses of light only when the incubator doors were opened. To study light-dependent degeneration, flies were exposed to light in an illuminated incubator at an intensity of 2000 lux. *rdgB*^*9*^, *P[w+,Rh1::GFP]*; *Rh1-Gal4*, *UAS-Dicer2* and *norpA*^*p24*^; *Rh1-Gal4*, *UAS-Dicer2* were the strains used for the genetic screens. GMR-Gal4 (second chromosome) was used for ERG experiments. UAS-RDGB, UAS-dCert::TurboID, 3HA-CG9205 and CKIIβ-3HA were used in co-IP experiments and generated in our laboratory.

### Fluorescent DPP analysis

Pseudopupil analysis was carried out on flies after days 2 and 4 post-eclosion. Flies were immobilized using a stream of carbon dioxide, and fluorescent pseudopupil analysis was carried out using an Olympus SZX12 stereomicroscope equipped with a fluorescent light source and GFP optics. Images were recorded using an Olympus digital camera.

### Optical neutralization

Flies were immobilized by cooling on ice. They were decapitated using a sharp razor blade and fixed on a glass slide using a drop of colourless nail varnish. The refractive index of the cornea was neutralized using a drop of immersion oil (*n* = 1.516 at 23°C); images were observed using a 40× oil-immersion objective (UPlanApo, 1.00 Iris; Olympus) with antidromic illumination (Franceschini & Kirschfeld, 1971). Images were collected on an Olympus BX-41 upright microscope and recorded using an Olympus digital camera.

### Electroretinogram recordings

Flies were anaesthetized and immobilized at the end of a disposable pipette tip using a drop of low melt wax. Recordings were done using glass microelectrodes filled with 0.8% wt/vol NaCl solution. Voltage changes were recorded between the surface of the eye and an electrode placed on the thorax. After fixing and positioning, flies were dark-adapted for 6 min. ERG was recorded with 1-s flashes of green light stimulus. Stimulating light was delivered from an LED light source within 5 mm of the fly’s eye through a fibre-optic guide. Voltage changes were amplified using a DAM50 amplifier (WPI) and recorded using pCLAMP 10.2. Analysis of traces was performed using Clampfit (Axon Laboratories).

### Co-immunoprecipitation (fly heads)

Fly heads with respective genotypes were lysed in ice-cold protein lysis buffer (50 mM Tris–HCl, 1 mM EGTA, 1 mM EDTA, 1% Triton X-100, 50 mM NaF, 0.27 M sucrose, and 0.1% β-mercaptoethanol). 10% of the lysate was aliquoted to be used as input. The remaining lysate was split into two equal parts. To one part, dVAP-A antibody (a kind gift from Girish Ratnaparkhi, IISER Pune) was added, and to the other part, a corresponding amount of control IgG (2729S; CST) was added, and incubated overnight at 4°C. On the next day, protein G Sepharose beads (GE Healthcare) were spun at 130,00*g* for 1 min and then washed with TBS twice. The beads were then incubated with 5% BSA (HiMedia) in TBS with 0.1% Tween-20 (TBST) for 2 h at 4°C. Equal amounts of blocked beads were then added to each sample and incubated at 4°C for another 2 h. The immunoprecipitates were then washed twice with TBST containing β-mercaptoethanol and 0.1 M EGTA for 5 min. The supernatant was then removed, and the beads were boiled in 2X Laemmli sample buffer for Western blotting. Primary antibodies used were as follows: rat anti-RDGB (laboratory-generated, 1:4,000), mouse anti-V5 (R960-25, 1:10,000; Invitrogen), and mouse anti-HA (1:1,000; CST). Respective secondary antibodies were used at the dilution of 1:10,000.

## Supplementary Material

Reviewer comments
