# Supplementary material for: A genetic screen to uncover mechanisms underlying lipid transfer protein function at membrane contact sites
Source: Life Sci Alliance. 2024 Mar 18;7(6):e202302525. doi: 10.26508/lsa.202302525 (PMC10948934; doi:10.26508/lsa.202302525)

| 1 | ***CG3401*** | 81 | ***CG8877*** | 161 | ***CG9543*** | 241 | ***CG11820*** | 321 | ***CG4396*** |
| --- | --- | --- | --- | --- | --- | --- | --- | --- | --- |
| 2 | ***CG9359*** | 82 | ***CG32075*** | 162 | ***CG1994*** | 242 | ***CG3542*** | 322 | ***CG6369*** |
| 3 | ***CG1828*** | 83 | ***CG33522*** | 163 | ***CG3491*** | 243 | ***CG4236*** | 323 | ***CG8954*** |
| 4 | ***CG2331*** | 84 | ***CG10415*** | 164 | ***CG44436*** | 244 | ***CG5585*** | 324 | ***CG16901*** |
| 5 | ***CG13900*** | 85 | ***CG9915*** | 165 | ***CG1871*** | 245 | ***CG15561*** | 325 | ***CG3860*** |
| 6 | ***CG2807*** | 86 | ***CG4087*** | 166 | ***CG2038*** | 246 | ***CG7162*** | 326 | ***CG2972*** |
| 7 | ***CG7626*** | 87 | ***CG17870*** | 167 | ***CG17446*** | 247 | ***CG3071*** | 327 | ***CG17489*** |
| 8 | ***CG10417*** | 88 | ***CG31196*** | 168 | ***CG3347*** | 248 | ***CG4488*** | 328 | ***CG3458*** |
| 9 | ***CG16742*** | 89 | ***CG9556*** | 169 | ***CG17440*** | 249 | ***CG8176*** | 329 | ***CG7516*** |
| 10 | ***CG8274*** | 90 | ***CG2173*** | 170 | ***CG1542*** | 250 | ***CG4364*** | 330 | ***CG43665*** |
| 11 | ***CG3312*** | 91 | ***CG1582*** | 171 | ***CG11444*** | 251 | ***CG44086*** | 331 | ***CG8414*** |
| 12 | ***CG6049*** | 92 | ***CG9323*** | 172 | ***CG4438*** | 252 | ***CG4049*** | 332 | ***CG18304*** |
| 13 | ***CG4299*** | 93 | ***CG32217*** | 173 | ***CG7686*** | 253 | ***CG6223*** | 333 | ***CG30170*** |
| 14 | ***CG5784*** | 94 | ***CG17437*** | 174 | ***CG8545*** | 254 | ***CG7538*** | 334 | ***CG8915*** |
| 15 | ***CG17593*** | 95 | ***CG10931*** | 175 | ***CG1276*** | 255 | ***CG7108*** | 335 | ***CG4379*** |
| 16 | ***CG3732*** | 96 | ***CG9594*** | 176 | ***CG10254*** | 256 | ***CG3335*** | 336 | ***CG10540*** |
| 17 | ***CG6227*** | 97 | ***CG8103*** | 177 | ***CG8635*** | 257 | ***CG11130*** | 337 | ***CG4878*** |
| 18 | ***CG2503*** | 98 | ***CG14884*** | 178 | ***CG11266*** | 258 | ***CG40494*** | 338 | ***CG5519*** |
| 19 | ***CG6708*** | 99 | ***CG9423*** | 179 | ***CG17768*** | 259 | ***CG42522*** | 339 | ***CG32707*** |
| 20 | ***CG1406*** | 100 | ***CG9205*** | 180 | ***CG6932*** | 260 | ***CG5642*** | 340 | ***CG6964*** |
| 21 | ***CG14472*** | 101 | ***CG17249*** | 181 | ***CG8863*** | 261 | ***CG31864*** | 341 | ***CG1430*** |
| 22 | ***CG34133*** | 102 | ***CG7061*** | 182 | ***CG12020*** | 262 | ***CG12264*** | 342 | ***CG33967*** |
| 23 | ***CG4548*** | 103 | ***CG6724*** | 183 | ***CG9828*** | 263 | ***CG15224*** | 343 | ***CG6701*** |
| 24 | ***CG10840*** | 104 | ***CG7597*** | 184 | ***CG11920*** | 264 | ***CG32505*** | 344 | ***CG6967*** |
| 25 | ***CG16941*** | 105 | ***CG33556*** | 185 | ***CG13667*** | 265 | ***CG3183*** | 345 | ***CG6379*** |
| 26 | ***CG4799*** | 106 | ***CG2890*** | 186 | ***CG13298*** | 266 | ***CG5904*** | 346 | ***CG13096*** |
| 27 | ***CG10986*** | 107 | ***CG7843*** | 187 | ***CG4119*** | 267 | ***CG3029*** | 347 | ***CG5728*** |
| 28 | ***CG7706*** | 108 | ***CG1433*** | 188 | ***CG8161*** | 268 | ***CG16792*** | 348 | ***CG8781*** |
| 29 | ***CG2469*** | 109 | ***CG10887*** | 189 | ***CG7207*** | 269 | ***CG9344*** | 349 | ***CG3949*** |
| 30 | ***CG2925*** | 110 | ***CG10418*** | 190 | ***CG6759*** | 270 | ***CG7917*** | 350 | ***CG5271*** |
| 31 | ***CG8548*** | 111 | ***CG4528*** | 191 | ***CG10811*** | 271 | ***CG9397*** | 351 | ***CG42341*** |
| 32 | ***CG12225*** | 112 | ***CG11418*** | 192 | ***CG10192*** | 272 | ***CG33106*** | 352 | ***CG4279*** |
| 33 | ***CG8892*** | 113 | ***CG1091*** | 193 | ***CG12372*** | 273 | ***CG6904*** | 353 | ***CG8069*** |
| 34 | ***CG12272*** | 114 | ***CG7163*** | 194 | ***CG5403*** | 274 | ***CG2257*** | 354 | ***CG11738*** |
| 35 | ***CG10080*** | 115 | ***CG8817*** | 195 | ***CG7467*** | 275 | ***CG14739*** | 355 | ***CG2253*** |
| 36 | ***CG5216*** | 116 | ***CG3889*** | 196 | ***CG9537*** | 276 | ***CG30342*** | 356 | ***CG16788*** |
| 37 | ***CG11990*** | 117 | ***CG5627*** | 197 | ***CG5800*** | 277 | ***CG2790*** | 357 | ***CG34334*** |
| 38 | ***CG7028*** | 118 | ***CG8427*** | 198 | ***CG2656*** | 278 | ***CG9124*** | 358 | ***CG8282*** |
| 39 | ***CG8400*** | 119 | ***CG1017*** | 199 | ***CG10222*** | 279 | ***CG10890*** | 359 | ***CG6905*** |
| 40 | ***CG1513*** | 120 | ***CG4697*** | 200 | ***CG9548*** | 280 | ***CG6315*** | 360 | ***CG1616*** |
| 41 | ***CG8725*** | 121 | ***CG8956*** | 201 | ***CG6554*** | 281 | ***CG4039*** | 361 | ***CG10370*** |
| 42 | ***CG7769*** | 122 | ***CG11654*** | 202 | ***CG6677*** | 282 | ***CG7006*** | 362 | ***CG11228*** |
| 43 | ***CG6222*** | 123 | ***CG9977*** | 203 | ***CG4806*** | 283 | ***CG6946*** | 363 | ***CG4063*** |
| 44 | ***CG31935*** | 124 | ***CG8625*** | 204 | ***CG1575*** | 284 | ***CG5824*** | 364 | ***CG4913*** |
| 45 | ***CG9484*** | 125 | ***CG7727*** | 205 | ***CG4886*** | 285 | ***CG5605*** | 365 | ***CG9635*** |
| 46 | ***CG1249*** | 126 | ***CG10161*** | 206 | ***CG1866*** | 286 | ***CG4817*** | 366 | ***CG5670*** |
| 47 | ***CG7728*** | 127 | ***CG4810*** | 207 | ***CG9916*** | 287 | ***CG3605*** | 367 | ***CG2747*** |
| 48 | ***CG5504*** | 128 | ***CG1815*** | 208 | ***CG8336*** | 288 | ***CG5654*** | 368 | ***CG11238*** |
| 49 | ***CG3909*** | 129 | ***CG5033*** | 209 | ***CG7768*** | 289 | ***CG5208*** | 369 | ***CG32146*** |
| 50 | ***CG13097*** | 130 | ***CG4202*** | 210 | ***CG17266*** | 290 | ***CG5229*** | 370 | ***CG3198*** |
| 51 | ***CG17255*** | 131 | ***CG10281*** | 211 | ***CG5258*** | 291 | ***CG3025*** | 371 | ***CG7971*** |
| 52 | ***CG8583*** | 132 | ***CG6538*** | 212 | ***CG31301*** | 292 | ***CG11290*** | 372 | ***CG5444*** |
| 53 | ***CG7757*** | 133 | ***CG30349*** | 213 | ***CG12608*** | 293 | ***CG1894*** | 373 | ***CG7015*** |
| 54 | ***CG10754*** | 134 | ***CG14444*** | 214 | ***CG9123*** | 294 | ***CG1691*** | 374 | ***CG13472*** |
| 55 | ***CG14894*** | 135 | ***CG1559*** | 215 | ***CG14805*** | 295 | ***CG7961*** | 375 | ***CG4539*** |
| 56 | ***CG3035*** | 136 | ***CG5786*** | 216 | ***CG2508*** | 296 | ***CG34126*** | 376 | ***CG11727*** |
| 57 | ***CG42665*** | 137 | ***CG33208*** | 217 | ***CG31687*** | 297 | ***CG7283*** | 377 | ***CG13185*** |
| 58 | ***CG8421*** | 138 | ***CG7035*** | 218 | ***CG3733*** | 298 | ***CG3843*** | 378 | ***CG32409*** |
| 59 | ***CG9351*** | 139 | ***CG7907*** | 219 | ***CG9253*** | 299 | ***CG4581*** | 379 | ***CG32211*** |
| 60 | ***CG8939*** | 140 | ***CG1554*** | 220 | ***CG42458*** | 300 | ***CG4622*** | 380 | ***CG10390*** |
| 61 | ***CG5659*** | 141 | ***CG4152*** | 221 | ***CG8092*** | 301 | ***CG9346*** | 381 | ***CG16973*** |
| 62 | ***CG4170*** | 142 | ***CG43658*** | 222 | ***CG10850*** | 302 | ***CG11271*** | 382 | ***CG2380*** |
| 63 | ***CG1677*** | 143 | ***CG32251*** | 223 | ***CG7041*** | 303 | ***CG1527*** | 383 | ***CG34401*** |
| 64 | ***CG40218*** | 144 | ***CG1427*** | 224 | ***CG15636*** | 304 | ***CG7036*** | 384 | ***CG3522*** |
| 65 | ***CG11427*** | 145 | ***CG2238*** | 225 | ***CG8120*** | 305 | ***CG33526*** | 385 | ***CG5589*** |
| 66 | ***CG9198*** | 146 | ***CG4849*** | 226 | ***CG8409*** | 306 | ***CG13849*** | 386 | ***CG1507*** |
| 67 | ***CG18332*** | 147 | ***CG1598*** | 227 | ***CG6990*** | 307 | ***CG10206*** | 387 | ***CG10528*** |
| 68 | ***CG8571*** | 148 | ***CG11985*** | 228 | ***CG10275*** | 308 | ***CG9018*** | 388 | ***CG4510*** |
| 69 | ***CG40351*** | 149 | ***CG11188*** | 229 | ***CG6349*** | 309 | ***CG31022*** | 389 | ***CG3725*** |
| 70 | ***CG33217*** | 150 | ***CG8610*** | 230 | ***CG4954*** | 310 | ***CG4164*** | 390 | ***CG7946*** |
| 71 | ***CG10223*** | 151 | ***CG7483*** | 231 | ***CG11417*** | 311 | ***CG6833*** | 391 | ***CG5434*** |
| 72 | ***CG5205*** | 152 | ***CG9075*** | 232 | ***CG2028*** | 312 | ***CG2791*** | 392 | ***CG17603*** |
| 73 | ***CG2093*** | 153 | ***CG7421*** | 233 | ***CG2048*** | 313 | ***CG9805*** | 393 | ***CG9062*** |
| 74 | ***CG3696*** | 154 | ***CG3817*** | 234 | ***CG9962*** | 314 | ***CG12792*** |  |  |
| 75 | ***CG6876*** | 155 | ***CG10955*** | 235 | ***CG7094*** | 315 | ***CG5004*** |  |  |
| 76 | ***CG5899*** | 156 | ***CG12498*** | 236 | ***CG2577*** | 316 | ***CG9983*** |  |  |
| 77 | ***CG32346*** | 157 | ***CG31703*** | 237 | ***CG12147*** | 317 | ***CG6354*** |  |  |
| 78 | ***CG7839*** | 158 | ***CG31702*** | 238 | ***CG2685*** | 318 | ***CG12749*** |  |  |
| 79 | ***CG1528*** | 159 | ***CG5794*** | 239 | ***CG7263*** | 319 | ***CG4262*** |  |  |
| 80 | ***CG14443*** | 160 | ***CG3730*** | 240 | ***CG6699*** | 320 | ***CG3151*** |  |  |


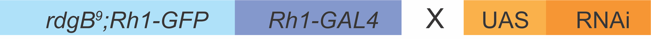


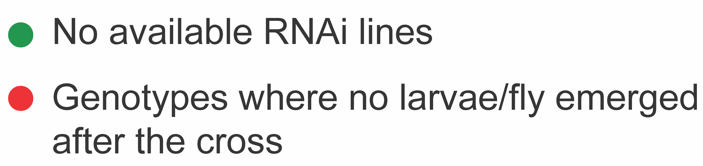

Supplement: Supplementary file 3 [file LSA-2023-02525_TableS3.docx]
